# Safety and Efficacy of Vitamin-based Antioxidant Therapy in Patients with Severe Acute Pancreatitis: A Randomized Controlled Trial

**DOI:** 10.4103/1319-3767.80379

**Published:** 2011

**Authors:** Dipika Bansal, Ashish Bhalla, Deepak K. Bhasin, Promila Pandhi, Navneet Sharma, Surinder Rana, Samir Malhotra

**Affiliations:** Department of Pharmacology, Postgraduate Institute of Medical Education and Research, Chandigarh, India; 1 Department of Internal Medicine, Postgraduate Institute of Medical Education and Research, Chandigarh, India; 2 Department of Gastroenterology, Postgraduate Institute of Medical Education and Research, Chandigarh, India

**Keywords:** Acute pancreatitis, antioxidant, free radicals, oxidative stress

## Abstract

**Background/Aim:**

Oxidative stress plays a major role in the pathogenesis of pancreatitis. Antioxidant therapy in the form of high-dose vitamin has been used for the treatment of severe acute pancreatitis with equivocal results. We wished to evaluate the efficacy and safety of antioxidant (vitamin A, vitamin C, vitamin E) therapy in patients with severe acute pancreatitis. Setting and design: This was a single-center, prospective, randomized, open-label with blinded endpoint assessment study of antioxidant therapy, conducted in the emergency department attached to our hospital.

**Materials and Methods:**

Thirty-nine patients with severe acute pancreatitis were randomly assigned to antioxidant treatment group (*n*=19) or a control group (*n*=20) within 96 hours of developing symptoms. Patients in the antioxidant group received antioxidants (vitamin A, vitamin E, vitamin C) in addition to the standard treatment provided to both the groups for a period of 14 days. The primary outcome variable was presence of organ dysfunction at day 7. The secondary outcome variables were length of hospital stay, multiorgan dysfunction (MODS) at day 7, recovery at the end of 4 weeks, complications, and mortality. The change in markers of oxidative stress from baseline was also measured.

**Results:**

We demonstrated no significant difference in organ dysfunction (*P*=1.0), MODS (*P*=0.8), and length of hospital stay (*P*=0.29) between the two groups. All the patients survived in the antioxidant-treated group, whereas two patients died in the control group. The change in the levels of malondialdehyde, superoxide dismutase, and reduced glutathione were not significantly different in the two groups at day 7. Univariate analysis showed marginal benefit with antioxidant treatment (*P*=0.034) in patients with severe acute pancreatitis.

**Conclusions:**

This randomized study demonstrates that there is no significant benefit from antioxidant therapy in patients with established severe acute pancreatitis.

Acute pancreatitis (AP) is predominantly secondary to symptomatic gallstone disease and excessive alcohol intake. Because of improvements in the management, including better diagnostics and treatment modalities, disease-related mortality has declined during the past two decades despite an increase in the overall incidence of AP in many countries. Most AP episodes are mild and self-limiting. However, about one-fifth of patients develop a severe acute pancreatitis (SAP), which is associated with a mortality rate of 40%. This type of AP is characterized by necrosis of the pancreas and surrounding tissue.[1]

Recent research has indicated that oxidative stress caused by short-lived intracellular oxygen-free radicals is one of the mediators of acinar injury in AP.[2] Oxidative stress occurs when oxygen-derived free radicals overwhelm endogenous oxidative defenses, causing damage to the cellular constituents. A number of experimental models[2] have shown that glutathione and other sulphydryl compounds are depleted and lipid peroxidation is increased in models of AP. Endogenous enzymic free radical scavengers are superoxide dismutase (SOD), catalase, myeloperoxidase (MPO), and glutathione peroxidase, and the non-enzymic scavengers are carotenes, ascorbic acid, and tocopherol.[2]

Beneficial effects of antioxidants and free radical scavengers have been shown in experimental models of AP.[3] Marked improvement in indices of pancreatic injury such as acinar cell injury and edema following treatment with enzymic antioxidants, vitamin C analogues, and glutathione monoethyl ester has been demonstrated. A few clinical studies are available;[4–11] notable amongst them is a small randomized control trial conducted by Siriwardena *et al*.[4]

Although these molecular and clinical studies provide a rational framework for studying the effect of antioxidant supplementation, there is limited data available on the effect of vitamin antioxidant supplementation in patients with SAP. The available clinical evidence is limited by studies including patients with mild disease and by heterogeneous definition of disease severity and complications. The lack of substantive evidence of benefit from antioxidant therapy in AP means that this treatment forms no part of contemporary guidelines for the management of this disease.[12]

The present study tries to evaluate the efficacy and safety of vitamin (A, C, and E) supplementation in patients with SAP as defined by computed tomography (CT) severity grading.

## MATERIALS AND METHODS

### Study design

This was a single-center, prospective randomized, open-label with blinded endpoint assessment study of antioxidant therapy, conducted over a period of 1 year in the emergency OPD attached to the Nehru hospital, PGIMER, Chandigarh, in patients with SAP.

### Inclusion/Exclusion criteria

We included in this study adult patients of either gender between 18 and 75 years of age presenting to the medical emergency unit, and whose clinical and biochemical presentation was consistent with AP (specifically, a history of acute abdominal pain associated with a greater than threefold elevation of the serum amylase and/or lipase >3 times normal, along with CT evidence of AP). Severe AP was defined for the purposes of this study as an APACHE II score of 8 or more at admission[13] and CT severity index (CTSI) ≥7.[14] Only those patients presenting with a first attack or an acute exacerbation of chronic pancreatitis were enrolled. The patients were enrolled within 96 hours of onset of the symptoms. All the criteria had to be present before the patient could be randomized. Exclusion criteria were: age <18 or >75 years; pregnancy; AP secondary to surgery, trauma, or malignancy; psychosis (except alcoholic delirium); need for urgent therapeutic intervention (endoscopic papillotomy, cholecystectomy, and/or choledochotomy); those enrolled in any other trial; patients with serious diseases of the heart, brain, liver, or kidney; peptic ulcer; autoimmune disease. The study was conducted after getting written permission from the institutional ethics committee. All the patients signed written informed consent before participating in the study.

### Standards of clinical care for SAP

The eligible patients were admitted to the medical emergency ward. A detailed history and general physical examination was carried out. CT of all the patients was done at admission to assess the severity of the condition. The standard basal treatment according to generally accepted principles and other optional treatment, if required, were given to all patients. Prophylactic antibiotics were used at the discretion of the treating clinician. Patients were followed up until discharge from hospital (or death).

### Study medication

In the present study, the patients were randomized (blocks of four) to two parallel groups: antioxidant group and control group. The antioxidant group received, in addition to maximal conventional therapy, vitamin C (1000 mg in 100 ml normal saline), vitamin E (200 mg oral), and vitamin A (10000 IU intramuscularly).The antioxidants were administered once daily. Initially the drugs were given parenterally (except vitamin E, as no parenteral formulation is available), followed by oral administration as soon as the patient’s condition permitted. The treatment was continued for 14 days from the day of enrolment into the trial. The control group was given the standard treatment only. The selection of doses was done according to previous studies.[7–13] Vitamin C was used in a megadose of 1 g/day, since there is evidence of early ascorbic acid depletion in patients with pancreatitis. The dose of vitamin A was sufficient to cure the deficiency without causing toxicity. The dose of vitamin E was also selected on the basis of previous studies.[7,9]

### Efficacy and safety assessment

The primary efficacy endpoint was the presence of organ dysfunction at day 7. The presence of organ dysfunction was determined as a score of ≥1 using the multiple organ dysfunction score (MODS).[15] The secondary efficacy endpoints were length of hospital stay in the two groups; proportion of patients recovering in each treatment group at day 7; MODS; change in the levels of oxidative stress markers [reduced glutathione (GSH), superoxide dismutase (SOD), malondialdehyde (MDA)] from baseline at day 7; and comparison of differences between the groups in the complication rate[16] at discharge. Laboratory parameters and clinically adverse events were monitored for safety analysis.

### Follow-up

The patients were monitored for complications [Table 1] on each day during the treatment phase (day 1 to day 14) and in the follow-up period till the day of discharge or till the end of 4 weeks, whichever was first. Theclinical and laboratory parameters were monitored for patient improvement.

**Table 1:** Complication score

Organ complication	Points
	
Shock	4
Sepsis	4
Pulmonary insufficiency	3
Renal insufficiency	3
Peritonitis	3
Hemorrhage	3
Abscess	3
Pseudocyst	3
Ileus/Subileus	1
Hypocalcemia	2
Coagulopathy	2
Hyperglycemia	2
Metabolic acidosis	2
Jaundice	1
Encephalopathy	1
Death	38

The complication score was assessed at enrolment and at the end of the follow-up period according to the scoring system described in Table 1.[16] The clinical change in each patient was calculated as the score on enrolment in the trial, minus the total score for newly developed complications during hospitalization. Thus, a zero or negative calculated value indicated clinical deterioration, while a positive value indicated clinical improvement. The final scoring was done at the time of discharge of the patient or at the end of 4 weeks, whichever was earlier.

### Measurement of oxidative and antioxidant markers

Three oxidative stress markers were measured in the patients at the time of enrolment of the patient into the trial and at day 7: reduced glutathione (GSH), superoxide dismutase (SOD) and malondialdehyde (MDA). Blood samples were collected at the appropriate time and centrifuged to separate plasma. The plasma was stored at -20°C till estimations were done according to the described procedures. All the chemicals used in the study were obtained from SRL Laboratories, CDH Labs, and Merck. The GSH levels were measured by spectrometry using sulfhydryl-dinitrobenzoic acid.[17] SOD was measured by spectrometry using the inhibitory effect of SOD on reduction of nitro blue tetrazolium.[18] MDA levels were measured by assaying the formation of thiobarbituric acid-reacting substance (TBARS).[19]

### Study conduct and data record

The study was conducted in an open-label manner. A separate investigator assessed each patient’s eligibility for trial participation and obtained the consent. The drugs were supplied according to the randomization schedule and were administered by the attending physician. Data was recorded prospectively by an independent investigator, using case report forms. An independent statistician, blinded to the intervention group, analyzed the data.

### Statistical analysis

The data is expressed as mean ± standard deviation, median with range, and as numbers and percentages. The presence of organ dysfunction was compared using Fisher exact test. The length of hospital stay was compared using Student’s unpaired *t* test, and proportions of patients improved were compared using the chi-square test. Oxidative stress markers were compared using Student’s t test. Difference in the complication scores at discharge were compared by Wilcoxon’s test and univariate analysis, with adjustment for baseline variability. Baseline parameters were compared by Student’s paired or unpaired *t*-test, Fisher exact test, and Mann-Whitney U test. A *P* value of less than 0.05 was considered statistically significant.

## RESULTS

**Figure 1:**
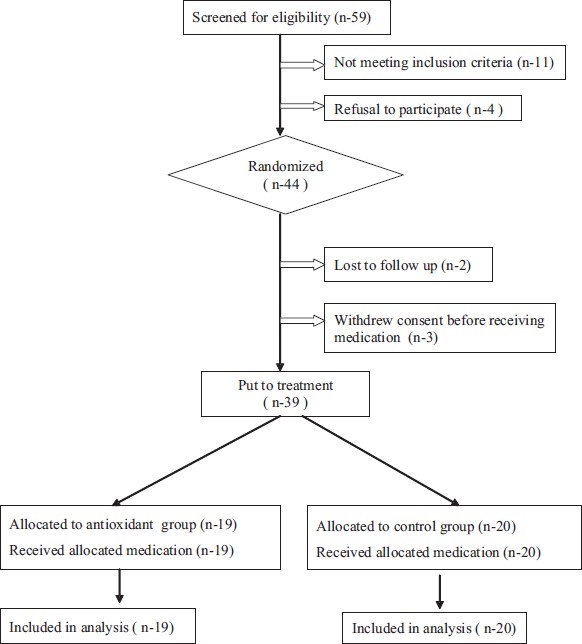
Consort flowchart of patients in the study

Patient recruitment started in April 2005 and the study was completed in May 2006. Patient disposition by treatment group is summarized in Figure 1 and the demographic and clinical characteristics are summarized in Table 2. A total of 59 patients were screened for the study and 48 patients were found eligible. Four patients refused to participate. Thus, 44 patients were randomized, out of which 39 completed the study. Baseline characteristics of patients showed an average age of 39 years (range 21–65). There were 30 (75%) males and 9 (25%) females [Table 2]. The etiology of AP was alcohol in 24 (62%) patients, gallstones in 7 (18%) patients, and other causes in 8 (20%) patients. The baseline demographic and clinical characteristics were comparable in both the groups.

**Table 2:** Baseline characteristics of trial participants

Characteristic	Antioxidant group(*n*=19)	Control group (*n*=20)	*P* value
Age (years)			
Mean±SD*	39.9 (10.9)	38.6 (11.4)	0.06
Median (range)	41.5 (21-57)	40 (24–65)	
Gender			
Males (%)**	15 (79)	15 (75)	1.00
Females (%)	4 (21)	5 (25)	
Presence of pain at randomization; no. (%)	19 (100)	20 (100)	-
Duration of pain before admission (days)			
Mean (SD)*	3.2 (1.4)	4.1 (1.9)	0.22
History of previous episode; no. (%)			
None**	12 (63)	11 (55)	0.71
1	4 (21)	4 (20)	
≥2	3 (16)	5 (25)	
Etiology; no. (%)			
Alcohol**	12 (64)	12 (60)	1.00
Gall stones	4 (21)	3 (15)	
Others	3 (14)	5 (25)	
CTSI (IQR)+	7 (7–9)	7 (7–8)	0.96
Days of intravenous treatment (SD)*	4.1 (2.5)	4.8 (2.1)	0.42
APACHE score (SD)*	11.2(2.9)	11.5 (2.7)	0.77
LODS at admission (IQR) +	3.1 (1–4)	3.4 (1–3.5)	0.90
MODS at admission (IQR) +	1.42 (0–2)	1.05 (0–2)	0.81

* Compared by Student’s unpaired *t* test

** Fisher exact test

+ Mann-Whitney U test. MODS: multi-organ dysfunction score; LODS: logistic organ dysfunction score; IQR: interquartile range

### Principal endpoints

The primary endpoint, i.e., presence of organ dysfunction at 7 days, was statistically similar in the antioxidant-treated and control groups (37% *vs* 40%) (*P*=1.0) [Table 3]. Analysis of MODS at day 7 revealed no significant difference between the two groups. The mean length of hospital stay in the antioxidant-treated group was 12.8 days vs 15 days in the control group. This was found to be statistically nonsignificant (*P*=0.29). The median duration for disappearance of pain in patients after inclusion into the study was 5 days (range: 2–8 days) in both the groups. By day 7, based on clinical and laboratory parameters, nine patients in antioxidant-treated group and six in the control group had improved (*P*=0.06). All the patients improved and were discharged in the antioxidant-treated group, whereas two patients in the control group died [Table 3].

**Table 3:** Baseline characteristics of trial participants

Characteristic	Antioxidant group(*n*=19)	Control group (*n*=20)	*P* value
Organ dysfunction# (day 7)	7 (37)	8 (40)	1.0
MODS (IQR)+	1.59 (0–3)	1.26 (0–3)	0.08
Length of hospital stay (days)			
Mean (SD)*	12.8 (3.9)	15.1 (5.43)	0.29
Median (range)	13 (9–21)	15 (3–22)	
Duration of pain, days			
Median (range)	5 (2–7)	5 (2–8)	-
Improvement at day 7 (based on clinical and laboratory parameters)#	9/14	6/15	0.06
Target organ dysfunction (day 7) Renal insufficiency#	4	4	1.0
Pulmonary insufficiency#	1	3	0.60
Abscess#	2	4	0.65
Difference in complication scores at outcome (SD)$	7.2 (12.9)	.71 (1.26)	.034
Laboratory markers (change from day 0 to day 7), mean (SD)*			
MDA (μmol/l)	–1.6 (1.9)	–1.5 (1.75)	6.4 (8.9)
GSH (mmol/l)	1.1 (0.7)	1.3 (1.7)	0.68
SOD (IU/dl)	5.4 (7.5)	0.64	0.74
Patient outcome,(%)			
Discharge	14 (100)	13 (86)	0.48
Death#	0 (0)	2 (14)	

$ Adjusted for differences at baseline, compared by Univariate analysis

* Student’s unpaired *t* test

# Fisher exact test

+ Mann-Whitney U test. MDA: malondialdehyde; GSH: reduced glutathione; SOD: superoxide dismutase

**Figure 2:**
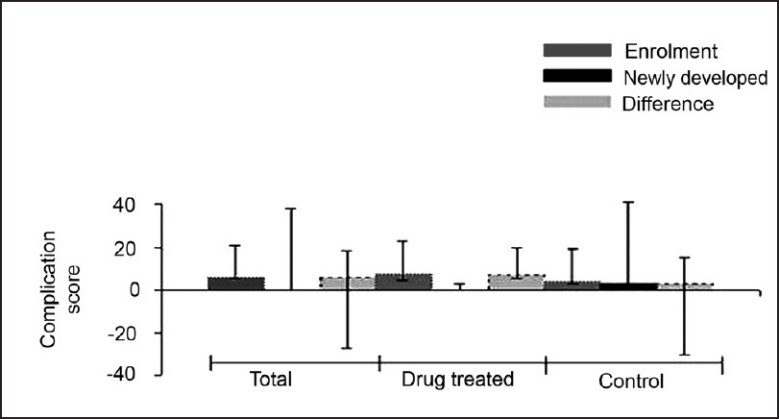
Complication scores on enrolment, newly developed complications, and the the difference between the two groups (results expressed as median and range)

Of the 39 patients who completed the study, 37 were discharged between days 3 to 22. Two patients died, both in the control group. Few patients developed new complications in the course of study. However, there was no significant difference among the two groups in terms of target organ dysfunction [Table 3] or in the frequency of complications [Figure 2]. The median complication score at day 0 in the antioxidant-treated group was 7.5 (range: 3–15) and in the control group it was 4 (range: 1–15). The median complication score at discharge/death in the treated group was 0 (range: 0–3) and in the control group it was 3 (range: 0–38). The median difference between the complication scores at day 0 and during the course of treatment (newly developed complications) was 7 (range: 2–13) in the treatment group and 7 (range: 3–12) in the control group. Though there was a baseline difference, the mean difference between the complication scores at inclusion and over the course of treatment (newly developed complications) was similar in both the groups. Univariate analysis (taking baseline variability into consideration) showed marginal improvement with the antioxidant treatment (*P*=0.034) [Table 3].

### Laboratory markers

No statistically significant difference in the levels of markers of oxidative stress (MDA, SOD, and GSH) were observed at baseline in both the groups. The levels of MDA were decreased and the levels of SOD and GSH were increased after 7 days in both the groups. The changes in the levels of these markers from baseline were not statistically significant in the two groups [Table 3].

### Adverse effects

There were no adverse effects directly attributable to antioxidant therapy. Close scrutiny of the two deaths in the control group revealed that both patients had coagulopathy at admission. One patient was 28 and the other 53 years old. Duration of disease from admission to death was 23 and 3 days, respectively. The probable cause of death was multiple organ failure secondary to SAP in both of them, but nosocomial infection may have contributed to demise.

## DISCUSSION

In the present study, we looked at the role of vitamin antioxidants in established SAP. In experimental AP, oxidative stress mediates (but does not initiate) acinar cell injury[2] and there is clinical evidence of serum antioxidant depletion during the course of AP.[20,21] There is little data available on the exact role of supplemental antioxidant vitamin therapy in high doses during the course of AP.

The presence of organ dysfunction and MODS at day 7 showed no significant difference in the two groups. Although the mean duration of hospital stay was decreased in the antioxidant-treated group, this did not reach statistical significance. A separate *post hoc* analysis was done excluding one patient in the control group who died early (on day 3), and the analysis then showed a trend towards a further decrease in the mean stay in hospital with the antioxidant supplementation (*P*=0.07). Two deaths in the control group and none in the treatment group suggests some benefit in the patients receiving antioxidants, but the number of patients in each group is too small to make any meaningful conclusions. Overall, there was little clinical improvement seen with antioxidant therapy in patients with SAP.

The increase in the levels of free radicals, as shown by raised levels of MDA at admission, was decreased on subsequent analysis at day 7 in both the groups. The depleted levels of SOD and GSH were restored to some extent late in the course of treatment in both groups, but the rise was not statistically significant post-supplementation in the treatment group. This, suggests that antioxidant administration did not significantly help in quenching free radicals generated during the inflammatory process of AP.

Our findings differ from that of an earlier study in which a high dose (10 g/day) of vitamin C was administered,[1] probably due to the relatively low dose (1 g/day) that we used. It might be postulated that stabilization of the disease naturally in due course, with conventional treatment and good ICU care, may have resulted in the decrease in oxidative stress markers in the samples collected at day 7 in both the groups.

The complication score was higher in the treatment group at inclusion. This was a chance finding. The median difference between the complication scores at inclusion and over the course of treatment (newly developed complications) was 7 in both the groups. The univariate analysis (taking the baseline variability into consideration) showed only marginal improvement with the treatment (*P*=0.034) in the antioxidant-treated group. There were few new complications that emerged in the intervention group as compared to the control group. The occurrence of two deaths in the control group may be a chance finding, or could have resulted from a higher baseline CTSI (early death) or development of nosocomial infection (late death). Although the difference in mortality between the two groups did not reach statistical significance it is clinically relevant, as antioxidant supplementation could modulate end organ injury and prevent mortality.

Current experimental evidence suggests that multiple antioxidant supplementation modulates end organ injury in experimental AP when intervention is done early in the disease course,[22] but not when intervention is delayed. Antioxidant therapy may be able to modulate mild to moderate injury but may not affect the more severe forms of disease, as seen in this study. Compared with an earlier cohort study, in which the median delay in instituting antioxidants was 2 (range: 0-7) days[13] and another randomized controlled study[10] in which the delay has not been mentioned, the antioxidant supplementation in the present study was administered with a median delay of 3 days (range: 1–8). The delay suggests that there was a well-established inflammatory process before the intervention. This is clinically relevant, since the concept of the ‘therapeutic window’[22] suggests that the best chance of success is by intervening at the disease inception. It is possible that the different outcomes in our study may also have been related to the time delay in instituting antioxidant therapy.

The trend for decreased hospital stay and development of fewer complications can translate into lower cost of hospitalization; however, a study conducted with a large sample size would be required to provide conclusive results. It is also possible that recruiting more patients may lead to a statistically significant fall in the mean duration of hospital stay, but then it may also result in increase in the number of new complications. The two deaths in the control group also carry clinical relevance, which could become statistically significant with an adequately powered study.

Our study has some limitations. This study was conducted over a 1-year period and the recruitment was not undertaken to satisfy a pre-specified power calculation but was limited to a planned recruitment period. As a result, the number of patients in both groups is small. The dose of vitamin C used in our study is also relatively low, but this may have been compensated for by the other two agents used as antioxidants. We could have studied the effect of individual antioxidants separately but for that a very large sample size would be needed, which was not feasible during the short study period. Also, the open-label nature of the study could have resulted in the introduction of some bias.

## CONCLUSIONS

The present study shows that vitamin-based antioxidant therapy has no significant beneficial effect on organ dysfunction or on clinical outcomes in severe acute pancreatitis during the hospital stay. Larger multicenter studies are required to address this issue in a more comprehensive manner.
